# Paired genomic and proteomic analysis of *Acinetobacter towneri* from waste sludge reveals an extensive repertoire of enzymes for breakdown of hydrocarbons

**DOI:** 10.1128/spectrum.00054-26

**Published:** 2026-03-05

**Authors:** Ronja Marlonsdotter Sandholm, Dave Rojas Calderón, Ørjan Dahl, Ravindra Reddy Chowreddy, Gustav Vaaje-Kolstad, Sabina Leanti La Rosa

**Affiliations:** 1 Faculty of Chemistry, Biotechnology and Food Science, Norwegian University of Life Sciences 201248 , Ås, Norway; 2 Norner Research AS 826124, Porsgrunn, Norway; Connecticut Agricultural Experiment Station, New Haven, Connecticut, USA

**Keywords:** plastics, low molecular weight polyethylene, ketones, alkanes, *Acinetobacter towneri*

## Abstract

Crude oil, primarily consisting of alkanes, and plastic derivatives are integral to modern society, yet their widespread use has led to persistent pollution that devastates ecosystems worldwide. Enzymatic and microbial biodegradation are highly desirable to become alternatives to conventional methods for the control of these pollutants in terrestrial and aquatic environments. Consequently, investigating the enzymatic machinery of specialized microbes that thrive in contaminated areas and possess the catabolic potential to degrade alkanes and plastics is of considerable interest. In this study, we present a genomic and proteomic analysis of *Acinetobacter towneri* RMS-02 that was isolated from sludge obtained from mixed food waste and sewage. Consistent with its ability to grow and persist in this environment, the genome of *A. towneri* RMS-02 encodes enzymes to catabolize different types of polyphenols and aromatic compounds, including *trans*-cinnamic acid, *o*-cresol, toluene, benzene, and benzoate. In addition, it encodes serine proteases, metallopeptidase, and transporters facilitating the uptake of amino acids and possibly small peptides, as well as enzymes potentially involved in the depolymerization of alkanes and 2-ketones. Proteomics analysis of *A. towneri* RMS-02 revealed an extensive repertoire of enzymes involved in terminal and subterminal oxidation of medium- and long-chain alkanes and ketones, which were specifically more abundant during growth on a product that consists of a mixture of these compounds and an oxidized low-molecular-weight polyethylene (LMWPE). Substrate characterization following bacterial growth confirmed the selective utilization of alkanes with chain lengths of C10–C25 and 2-ketones of C13–C26, while the polymeric fraction of the substrate remained unaltered.

**IMPORTANCE:**

Crude oil and plastic pollution threaten ecosystems worldwide, creating an urgent need for sustainable remediation strategies. Microbial and enzymatic degradation provides a sustainable alternative to physical, chemical, and thermal treatments by biologically breaking down hydrocarbons into harmless products or recyclable monomers. Here, we describe a detailed genomic characterization of *A. towneri* RMS-02, identifying core metabolic functions underlying its potential to utilize phenols, aromatic compounds, proteins, and small-chain hydrocarbons. Using proteomics, we show that a diverse enzymatic arsenal is deployed when growing on an oxidized LMWPE product that includes a mixture of alkanes and 2-ketones. Remarkably, the proteomics results were corroborated by advanced analysis of the spent substrate, confirming that *A. towneri* RMS-02 metabolizes alkanes and 2-ketones but is unable to interact with the polymeric LMWPE component. Our results expand the understanding of the metabolic repertoire supporting *Acinetobacter towneri*’s survival and identify candidate enzymes with potential for the bioremediation of alkanes and 2-ketones.

## INTRODUCTION

Long-chain alkanes, defined as hydrocarbons with more than 16 carbon atoms (1), pose environmental concerns as well as complicate crude oil handling by clogging pipelines and hindering recovery, transport, and processing (2). These alkanes and their oxidized derivatives, such as 2-ketones, can also be released through chemical, thermal, and photo-oxidation of polyethylene, one of the most widely produced and environmentally persistent synthetic polymers (3–5). Microbes capable of degrading hydrocarbons might offer a reservoir of enzymes, which can transform these pollutants into more valuable or manageable products. In this context, environments contaminated with alkanes or crude-oil-derived plastics can represent a reservoir of microorganisms that have adapted or evolved to exploit these compounds. To date, the genus *Acinetobacter* has emerged as a versatile degrader, with several strains reported to effectively break down diverse pollutants, such as alkanes, pesticides, plasticizers, and possibly plastics, highlighting the genus’s broad potential for bioremediation (6, 7). Specifically, *Acinetobacter* sp. strain DSM 17,874 and strain M-1, both isolated from oil-contaminated soils, can utilize C10–C44 alkanes (8, 9). *Acinetobacter vivianii* KJ-1, from a diesel-contaminated environment, degrades C10–C12 alkanes (10). *Acinetobacter* spp. RAG-1, BT1A, and AR-46, also from oil-contaminated soils, target long-chain alkanes, while *Acinetobacter sp*. GJ70 degrades various aliphatic and halogenated alkanes (11–13). *Acinetobacter baylyi* ADP1, isolated from soil, has been shown to metabolize C12–C36 alkanes (11).

Over the past several decades, studies on alkane degradation have primarily focused on identifying and characterizing enzymes that catalyze the initial steps of aerobic bacterial catabolism. Two different pathways have been described. In the terminal oxidation pathway, alkanes are oxidized to their corresponding primary alcohols by substrate-specific terminal monooxygenases or hydroxylases, such as AlkM, AlkB, and cytochrome P450, including CYP153 (14). The resulting primary alcohol is further sequentially oxidized by an alcohol dehydrogenase and an aldehyde dehydrogenase to form the corresponding aldehyde and carboxylic acid, respectively. The carboxylic acid then serves as a substrate for acyl-CoA synthetase, resulting in acyl-CoA, which enters the β-oxidation pathway. Additionally, a subterminal oxidation pathway has also been described, not only for long-chain alkanes but also for medium-chain alkanes (C10–C16). Here, the subterminal carbon of the alkane is hydroxylated by monooxygenases, such as LadA or AlmA, forming a secondary alcohol, which is subsequently converted into a ketone by alcohol dehydrogenases and further processed by Baeyer–Villiger monooxygenases and esterases to generate acetate and a primary alcohol (15).

Among the *Acinetobacter* species, *Acinetobacter towneri* was first isolated from activated sludge in Bendigo, Australia, and has since been characterized for its environmental and clinical significance (16). Different strains have been studied for their potential in bioremediation, particularly in degrading phenolic compounds. As an example, the strain *A. towneri* CFII-87, isolated from a bioreactor treating landfill leachate, was shown to grow on and metabolize phenol up to 1,000 mg/L concentration, with maximum growth at 300 mg/L. In bioaugmentation experiments, *A. towneri* CFII-87 achieved over 98% phenol removal within 16 h in phenol-rich synthetic wastewater (17). Despite the promise of *A. towneri* for bioremediation in contaminated environments, *A. townerii* strains capable of metabolizing alkanes or polyethylene-containing plastics remain scarce, and their enzymatic machinery is poorly characterized. Notably, to date, no functional studies, with either transcriptomics or proteomics, have investigated if and how *A. townerii* strains respond to alkanes and oxidized variants thereof.

In this study, a novel strain of *A. townerii*, RMS-02, was isolated from waste sludge. We conducted a genomic and proteomic analysis to determine the ability of RMS-02 to metabolize key components in its source environment, including phenols, proteins, and small-chain hydrocarbons, enabling its growth and persistence. Additionally, we investigate the molecular principles of alkanes and 2-ketone utilization and suggest enzyme candidates for bioremediation of these compounds. Our results significantly widen the recognized metabolic capacity of the species, highlighting *A. towneri* as a previously underrecognized participant in the microbial transformation of environmental contaminants.

## MATERIALS AND METHODS

### Sample collection

Samples were collected in September 2023 from a 2,200 m^3^ thermophilic biogas plant (FrBGR) in Fredrikstad, Norway. This plant has been operating stably for a decade at 60°C, mainly using food waste and sewage sludge as the substrate for producing biogas. Here, grounded food waste and homogenized sewage sludge are combined, pasteurized at 70°C, and then pumped into three series-connected rotting tanks for anaerobic decay. A sample was collected from the sludge released from the last rotting tank, frozen in 20% (vol/vol) glycerol, and stored at −80°C until further processing.

### Enrichment and isolation of *Acinetobacter towneri* RMS-02

Enrichment cultures were set using a minimal salts medium (MM) supplemented with 10 mg/mL of an oxidized low-molecular-weight PE (LMWPE, Sigma-Aldrich, 427772), a substrate known to consist of alkanes and 2-ketones (chain length of 10–34 carbons), as well as an oxidized polymeric component (chain average carbon chain length of 279) (18). Each liter of MM (pH 7.2) contained 10% 10× M9 medium (0.56 M Na_2_HPO_4_, 0.29 M KH_2_PO_4_, 85.56 mM NaCl, and 93.47 mM NH_4_Cl), 1% (vol/vol) 100× trace elements solution (17.11 mM, pH 7.5, 3.07 mM FeCl_3_× 6H_2_O, 0.62 mM ZnCl_2_, 76.26 µM CuCl_2_× 2H_2_O, 42.03 µM CoCl_2_× 6H_2_O, 0.16 mM H_3_BO_3_, and 8.08 µM MnCl_2_ × 4H_2_O), 0.1% (vol/vol) 1 M MgSO_4_, 0.03% (vol/vol), 1 M CaCl_2_, 0.1% (vol/vol), 1 mg/mL biotin, and 0.1% (vol/vol) 1 mg/mL thiamin. A flask with 20 mL of MM supplemented with LMWPE was inoculated with 200 μL of glycerol stock from the biogas facility and incubated for 48 h at 30°C and 200 rpm to obtain a primary culture. Two additional passages were performed by sequentially transferring 50 μL of the primary and secondary cultures into fresh 5 mL MM supplemented with oxidized LMWPE and incubating under the same conditions as above. After that, the enrichment culture (designated as tertiary culture) was plated onto LB agar plates that were incubated for 72 h at 30°C. Individual colonies were transferred back to MM containing 10 mg/mL LMWPE and grown overnight. Positive cultures were then plated onto LB agar plates and incubated for 2 days at 30°C. Single colonies (*n* = 8) were dissolved in 20 µL dH_2_O, and 2 µL was used as template DNA with 2.5 µL 10 µM forward primer ([27F] 5′-AGAGTTTGATCMTGGCTCAG-3′), 2.5 µL 10 µM reverse primer ([1492R] 5′-GGTTACCTTGTTACGACTT-3′), 25 µL Q5 High-Fidelity 2X Master Mix (New England Biolabs, cat. number: M0492S), and 18 µL dH_2_O. The 16S rRNA gene was amplified using the following program: initial denaturation at 98°C for 2 min, 35 cycles of amplification (98°C for 10 s, 55°C for 30 s, 72°C for 50 s), and a final elongation at 72°C for 10 min. The PCR products were cleaned using the Nucleospin Gel and PCR Clean-up kit (Machery-Nagel, Germany), following the manufacturer’s protocol. DNA concentration and purity were evaluated using a NanoDrop One Microvolume UV-Vis spectrophotometer (ThermoFisher Scientific, CAT: ND-ONE-W). The amplicons were sent to Eurofins Genomics (Moss, Norway) and sequenced using Sanger sequencing. Based on the results, all the colonies were taxonomically assigned to the species *Acinetobacter towneri*. A single isolate was randomly selected for further study and designated *A. towneri* RMS-02.

### Whole-genome sequencing, assembly, and annotation

*A. towneri* RMS-02 was cultured in BHI at 30°C overnight, and genomic DNA was extracted using the DNeasy PowerSoil Pro Kit (QIAGEN) following the manufacturer’s instructions. DNA concentration was measured using a NanoDrop One spectrophotometer and a Qubit 3.0 fluorometer with the dsDNA High Sensitivity Assay Kit (Thermo Fisher Scientific). DNA quality was assessed by gel electrophoresis on a Bio-Rad Gel Doc EZ Imager. A sequencing library was prepared using the Native Barcoding kit SQK-NBD114.24 following the manufacturer’s protocols. The library was loaded onto a FLO-MIN114 R10.4 flow cell and sequenced for 48 h on a MinION device using MinKNOW v4.0.5. POD5 files were basecalled and demultiplexed with Dorado v0.5.0 using the super-accurate model (dna_r10.4.1_e8.2_400bps_sup@v4.3.0). Raw reads were quality-filtered with FiltLong v0.2.1 (https://github.com/rrwick/Filtlong) using the parameters --min_length 3000, --keep_percent 90 and assembled using Flye v2.9.2 (--nano_hq, --min-overlap 1500, default settings) (19). Contigs were initially polished with two consecutive rounds using Medaka v2.0.1 (https://github.com/nanoporetech/medaka) with standard parameters, followed by mapping raw reads to the consensus sequence using minimap2 v2.28-r1209 (20). The alignment was polished using Racon v1.5.0 (-m 8 -x -6 -g -8 -w 500) (https://github.com/isovic/racon). Assembly completeness was evaluated with BUSCO v6.0 (21) (-m geno, --auto-lineage-prok) and CheckM2 v1.1.0 (22). GTDB-Tk v 2.4.1 (23) with GTDB release 220 was used to determine the taxonomic assignment. Functional annotations were obtained with DRAM v1.5.0 (24). Carbohydrate-active enzymes and enzymes involved in the utilization of polyphenols were predicted using CAZyLingua (25) and CAMPER (26), respectively. To assess the genome for the presence of genes encoding enzymes involved in protein and peptide degradation, as well as transporters for uptake of amino acids and peptides, an in-house database of genes and associated EC numbers was blasted against the *A. towneri* RMS-02’s genome. The genome was considered positive for a given function if its genes exhibited ≥75% nucleotide identity and ≥80% coverage relative to the reference sequence. To predict the subcellular localization of the detected predicted proteins, we used SignalP (version 6.0; default settings for Gram-negative bacteria) to identify signal peptides and their predicted cleavage by signal peptidase I (SpI) or signal peptidase II (SpII). Proteins were classified as secreted if such signal peptides were detected.

### Phylogomic tree

For the phylogenomic analysis of *A. towneri* RMS-02, a list of 58 *Acinetobacter* species was compiled using their GTDB species representatives and NCBI type material (Table S1a). The publicly available genome sequences of the bacteria were downloaded from NCBI. Taxonomic classification of all genomes was conducted using GTDB-Tk v2.4.0 (23) with the GTDB release 224. Phylogenetic relationships were inferred from a concatenated alignment of 120 conserved single-copy proteins identified using GTDB-Tk. Maximum-likelihood tree construction was performed on the alignment using IQ-TREE (27) with the following settings ‘-m MFP -bb 1000 -nt 16’. The resulting tree was rooted with *Moraxella catarrhalis* CCRI-195ME (GCF_002080125.1) as the outgroup and visualized in R v4.3.3 (28) with the packages tidyverse v2.0.0 (29), ggtree v3.10.0 (30), tidytree v0.4.6 (31), and ape v5.8.1 (32).

For *A. towneri* RMS-02 and *A. towneri* DSM14962, ANI values were calculated using FastANI v1.34 (33). AAI values were determined using FastAAI v0.1.20 (34).

### Genomic potential for the degradation of fossil-fuel-based and renewable polymers

The genome of *A. towneri* RMS-02 was screened for 593 unique number gene products associated with the degradation of diverse synthetic and renewable polymers, as well as aliphatic hydrocarbons. The list of these genes was compiled from the PlasticDB database (35), the Plastics-Active Enzymes Database (PAZy) (36), and the Hydrocarbon Aerobic Degradation Enzymes and Genes (HADEG) database (37). The target polymers considered in this search included PE, oxidized derivatives of PE (alkanes and 2-ketones), polyethylene terephthalate (PET) and polyurethane (PU), polyamide (PA), polystyrene (PS), polylactic acid (PLA), polyhydroxyalkanoates (PHA), polyhydroxybutyrate (PHB), polybutylene adipate terephthalate (PBAT), polybutylene succinate (PBS), and natural rubber (NR). The protein sequences of the 593 plastic- and hydrocarbon-degradation genes were used as a query to search the subject database using Protein-Protein BLAST v2.16.0+ (38) with settings “-max_target_seqs 593” (number of sequences in the query data set). BlastP hits were filtered for ≥50% identity (pident), ≥75% query coverage (qcovhsp), and e-value of ≤1.0e-5. For each protein, the best hit was retained. The annotated genome was also searched for enzymes known to be involved in alkane and fatty acid degradation, based on KEGG annotations (39).

### Growth experiments

Growth experiments were carried out in MM supplemented with 30 mg/mL of either an oxidized low-molecular-weight PE (LMWPE, Sigma-Aldrich, 427772) or sodium succinate (Sigma-Aldrich, S2378). A 200 µL aliquot of overnight culture was used to inoculate 20 mL of MM plus the substrate to be tested. These precultures were sub-cultured at least three times on the same medium to allow cells to adapt to the specific single carbon source before inoculating the final cultures used for growth profiling and proteomic analysis. Growth was assessed by measuring the optical density (absorbance) at 600 nm (OD_600_) at regular intervals for 7 days. Growth curves represent the mean of two biological replicates, each with three technical replicates.

### Proteomic analysis

*A. towneri* RMS-02 was grown in triplicate on MM supplemented with either 30 mg/mL sodium succinate or LMWPE, respectively, as the sole carbon source. Samples (20 mL) were harvested at the mid-exponential growth phase. LMWPE particles were collected from the liquid culture by filtration using Whatman 20 µm cellulose filters (Cytiva, USA; Cat. No: WHA10331554). Biofilm material was detached from the collected particles by ultrasonication in a lysis buffer (20 mM Tris-HCl, pH 7.5; 100 mM NaCl; 1% SDS; 1 mM EDTA). After centrifugation at 4,500 × *g* for 10 min at 4°C to remove debris, the clarified supernatant was up-concentrated using 3 kDa Vivaspin 20 filters (3-kDa molecular weight cutoff, Sartorius Stedim Biotech GmbH, Germany) to obtain biofilm-derived proteins for further analysis. Planktonic cells were recovered from the resulting LMWPE-free liquid cultures by centrifugation (4,500 × *g*, 10 min, 4°C), resuspended in 50 mM Tris-HCl, pH 7.5, 100 mM NaCl, 0.1% (vol/vol) Triton X-100, 1 mM dithiothreitol (DTT), and disrupted by bead-beating (three 60 s cycles) with a FastPrep24 (MP Biomedicals, CA) at 6.5 m/s. Biofilm- and planktonic cell-derived proteins were precipitated with 10% (vol/vol) ice-cold trichloroacetic acid (TCA), incubated overnight on ice, centrifuged (15,000 × *g*, 15 min, 4°C) to pellet the precipitated proteins, and washed with 300 μL ice-cold 0.01 M HCl in 90% acetone. After removal of the supernatant, the protein pellets were air-dried and resuspended in 46 µL 1× SDS lysis buffer (5% SDS and 50 mM triethylammonium bicarbonate, pH 8.5). Protein digestion was performed using S-Trap Mini Columns (Protifi, Fairport, NY, USA), according to the manufacturer’s instructions, with 20 mM DTT for reduction and 40 mM iodoacetamide for alkylation. Peptides were analyzed on a nanoLC-MS/MS system consisting of a nano UHPLC (nanoElute 2, Bruker Daltonics Inc., Bremen, Germany) coupled to a trapped ion mobility spectrometry–quadrupole time-of-flight mass spectrometer (timsTOF Pro, Bruker Daltonics Inc., Bremen, Germany). Separation was achieved on an Aurora C18 reverse-phase (1.6 µm, 120 Å) 25 cm × 75 µm analytical column with an integrated emitter (IonOpticks, Melbourne, Australia). Mass spectral data were acquired using DataAnalysis v6.1.

MS raw files were processed using the FragPipe v21.1 (with MSFragger v4.0, IonQuant v1.10.12, Philosopher v5.1.0, DIA-NN v1.8.2 beta 8) (40) for protein identification and label-free quantification (LFQ). MS and MS/MS spectra were searched against the complete proteome of *Acinetobacter towneri* RMS-02 (2,602 proteins), supplemented with common contaminants (e.g., keratins, trypsin, and bovine serum albumin) using a decoy database consisting of reversed sequences of all protein entries for false discovery rates (FDRs) estimation. The resulting protein library consisted of 5,440 proteins. Trypsin was set as the proteolytic enzyme, with one missed cleavage allowed. Variable modifications included methionine oxidation and pyro-glutamate formation at N-terminal glutamines, while carbamidomethylation of cysteines was set as a fixed modification. Protein identifications were filtered to achieve a 1% FDR. A protein was considered “present” if detected in at least two of the three biological replicates for at least one substrate. Missing values were imputed from a normal distribution (width: 0.3; downshift: 1.8 standard deviations from the original distribution) using the “total matrix mode.” Differential abundance analysis was conducted with Rstatix v0.7.2 (41) using a paired Student’s *t*-test with a permutation-based FDR threshold set at 0.05. Figures were generated using R v4.3.3 (28) with the packages ggplot2 v3.5.1 (42) and cowplot v1.1.3 (43).

### Assessment of LMWPE degradation

Two analytical techniques were employed to characterize structural and compositional changes in LMWPE resulting from the growth of *A. towneri* RMS-02 (*At*LMWPE). As a control, untreated LMWPE (uLMWPE) and LMWPE incubated in a cell-free MM (cfLMWPE) were included. To determine changes in the size of the polymer chains, Size Exclusion Chromatography (SEC) was performed using a GPC-IR5 system (Polymer Char, Valencia, Spain). About 4 mg of each sample was dissolved in 8 mL of 1,2,4-trichlorobenzene at 160°C for 3 h. A 200 µL aliquot was injected into the SEC system equipped with four PLgel 20 µm MIXED-A columns (Agilent Technologies, Santa Clara, CA, USA). Analyses were performed at 150°C with 1,2,4-trichlorobenzene as the mobile phase (flow rate: 1 mL/min) and a high-sensitivity infrared detector. Calibration was performed using narrow-distribution polystyrene standards with peak molecular weights (*M*_peak_) from 1,140 to 7,500,000 g/mol. The weight-average molecular mass (*M*_*w*_), number-average molecular mass (*M*_*n*_), and molecular weight distribution were determined. Gas Chromatography–Mass Spectrometry (GC-MS) was used to quantify changes in the amounts of low-molecular-weight compounds using an Agilent 6890N GC with a 5973 MS detector and a GERSTEL MPS2 autosampler. Approximately 100 mg of each polyethylene sample and 3 mL of ethyl acetate were transferred into 4 mL glass vials, sealed, and the extractable components were extracted at 65°C for 1.5 h. Extracts were filtered (0.45 µm Teflon syringe filter) before injection. Separation was achieved on a Zebron ZB-5MSPlus column (30 m × 250 µm, 0.25 µm film) with oven programming from 60°C to 300°C at 10°C/min (total 45 min). Helium (grade 6.0, 3 mL/min) served as a carrier gas. Injections were splitless at 250°C; ion source temperature was 230°C; and electron ionization was performed at 70 eV. Spectra were collected over m/z 33–720. Each sample was analyzed in triplicate (*n* = 3), and compounds were identified against the Wiley 11th/(NIST 2017) library.

## RESULTS AND DISCUSSION

### Whole-genome sequencing and annotation of *A. towneri* RMS-02

**Fig 1:**
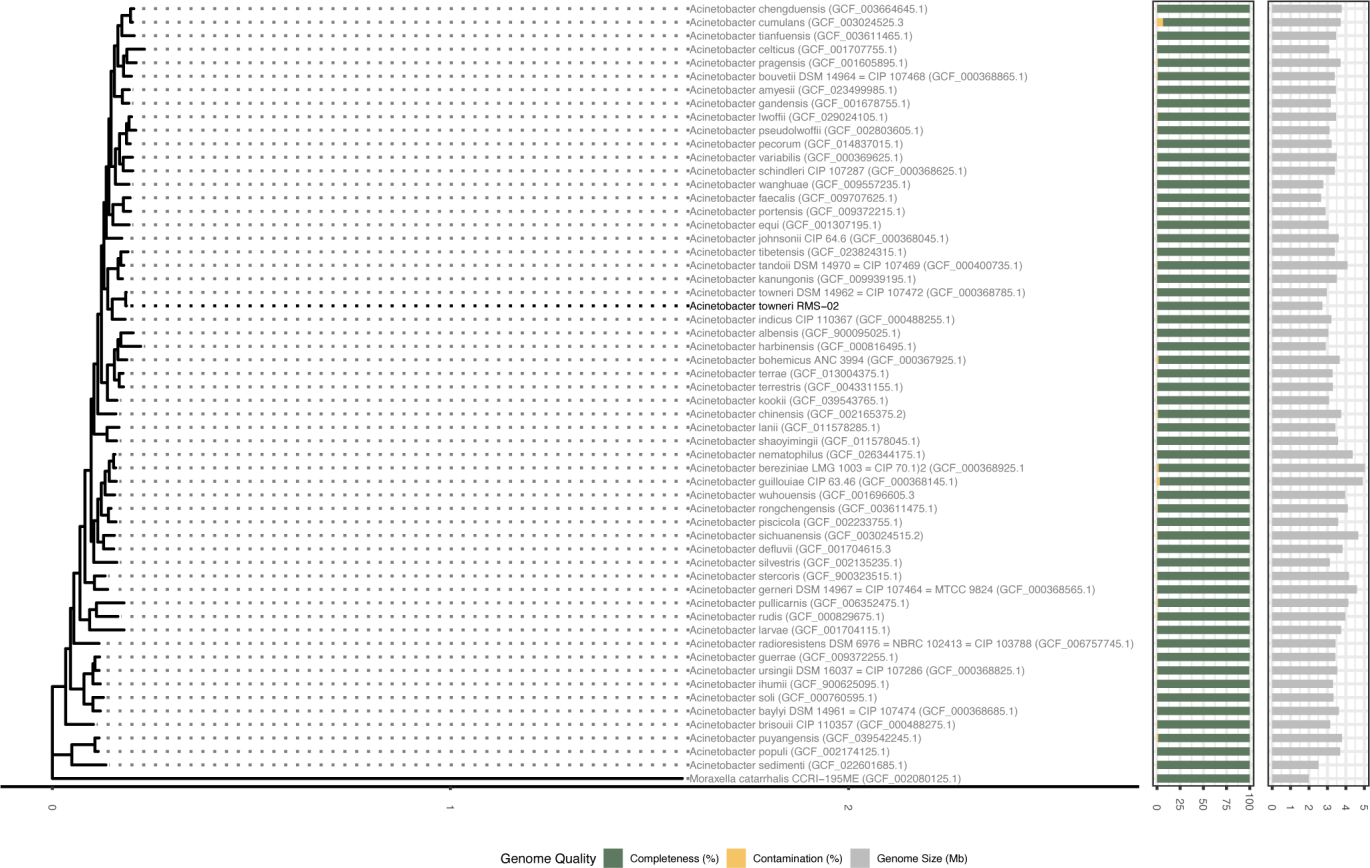
Phylogenetic tree inferred from a concatenated alignment of 120 conserved single-copy gene sequences, including *A. towneri* RMS-02 alongside 58 type strains of *Acinetobacter* spp. *Moraxella catarrhalis* CCRI-195ME served as the outgroup. Green and yellow bars indicate genome completeness and contamination (%), respectively. The gray bars indicate the genome size (in Mb). The scale bar at the bottom of the phylogenetic tree indicates the expected number of substitutions per amino acid site.

In an effort to identify microorganisms and their enzymes capable of depolymerizing environmental contaminants, such as long-chain alkanes and plastics, *A. towneri* RMS-02 was isolated from the rotting tank of a biogas facility in Norway using an enrichment culture strategy based on the ability of microbes to grow on a substrate consisting of a mixture of alkanes, 2-ketones, and oxidized LMWPE. The isolation strategy included three sequential passages of the culture in minimal medium supplemented with this substrate as the sole carbon source. Following this, the tertiary culture was plated on LB agar, and growth of eight selected colonies was verified in minimal medium containing the same initial substrate mixture. Colonies were taxonomically identified via 16S rRNA gene amplification, and a single isolate, designated RMS-02, was randomly selected for subsequent genomic and proteomics studies. The *A. towneri* RMS-02’s genome was sequenced using long-read sequencing with Oxford Nanopore technology, generating a total of 2.06 Gb of raw DNA sequences. The data were assembled into 2 contigs, with an N50 of 2,669,569 bp. The total genome length was 2,722,863 bp, with the longest contig measuring 2,669,568 bp and a plasmid of 53,296 bp, both predicted to be circular. The overall G+C content was 41.39%. The chromosome achieved a coverage of 167×, while the plasmid achieved a coverage of 162×. Genome quality assessments with CheckM2 (22) and BUSCO (21) indicated 100% completeness and 1.9% contamination. In total, 2,548 genes, 77 tRNAs, and 21 rRNAs (including 7 16S rRNA, 7 23S rRNA, and 7 5S rRNA) were predicted. A phylogenomic tree was constructed based on the alignment of 120 genes from GTDB positioned *A. towneri* RMS-02 as the closest relative of *A. towneri* DSM14962 (Fig. 1), an isolate sourced from activated sludge (16). Of note, *A. towneri* RMS-02’s genome is smaller compared to that of *A. towneri* DSM14962, being 2.7 Mb and 3 Mb, respectively. The ANI and AAI value between *A. towneri* RMS-02 and *A. towneri* DSM14962 are 97.65% and 99.19%, respectively. While the AAI and ANI values are within the species cutoff, the ANI value indicates the status of novel strain (44).

### Genome insights into the predicted machinery of *A. towneri* RMS-02 to metabolize glycans, polyphenols, and short hydrocarbons

The organic fraction of wastewater and sewage primarily consists of plant-derived materials (cellulose, hemicellulose, and lignin), phenolic compounds, proteins, and lipids derived from food residues and human waste. In addition, microplastics, small plastic particles less than 5 mm in size, can persist throughout treatment processes (45, 46). We examined the predicted ability of *A. towneri* RMS-02 to use these components to support its growth. First, the genome was annotated with dbCAN3 (within DRAM [24]) and CAZyLingua (25) to investigate carbohydrate-active enzyme family repertoires, specifically the presence of glycoside hydrolases (GH), carbohydrate esterases (CE), polysaccharide lyases (PL), and auxiliary activities (AA) (Table S1b and c). No families potentially enabling the utilization of cellulose and hemicelluloses were detected. Instead, the only identified GHs were predicted to be peptidoglycan-lytic β-1,4-muraminidase, belonging to the families GH19, GH23, GH25, GH103, GH153 as well as β-*N*-acetylhexosaminidase GH3, all of which are known to be implicated in cell-wall turnover. The genome also encodes a laccase/p-diphenol:oxygen oxidoreductase AA1, a vanillyl-alcohol oxidase AA4, and two P-benzoquinone reductase AA6, potentially involved in metabolism of lignin-derived monomers or oxidized derivatives.

Polyphenols are bioactive compounds naturally found in plants, including polymers (such as tannins), monomers (e.g., flavonoids), and simple phenols (e.g., phenolic acids) (47). To identify genes encoding enzymes involved in the utilization of diverse polyphenols, we employed the software CAMPER (see Materials and Methods). CAMPER assigns genes to 30 established polyphenol classes and organizes them into 100 metabolic transformation pathways based on biochemically characterized homologs (26). *A. towneri* RMS-02’s genome included a complete set of genes for trans-cinnamate degradation (Table S1d), such as *hcaD*, *hcaE*, *hcaF*, *hcaC*, and *hcaB*, which encode components of the 3-phenylpropionate/trans-cinnamate dioxygenase complex (EC:1.14.12.19 and EC:1.18.1.3), as well as downstream enzymes like *mhpA*, *mhpB*, *mhpC*, *mhpD*, and *mhpE* involved in hydroxylation, ring cleavage, and conversion to metabolites feeding the tricarboxylic acid (TCA) cycle. Similarly, the phenylpropionate pathway was represented by homologous genes (*hpaD*, *hpaE*, *hpaF*, *hpaC*) and associated dehydrogenases and hydratases (Table S1d), enabling oxidation and cleavage of 3-phenylpropionic acid into acetyl-CoA and succinyl-CoA, which can then enter the TCA cycle for energy generation. For catechol metabolism, the *A. towneri* RMS-02 genome contains genes for enzymes catalyzing the ortho-cleavage route, commonly referred to as the β-ketoadipate pathway. This pathway includes *catA* (catechol 1,2-dioxygenase, EC:1.13.11.1), *catB* (muconate cycloisomerase), *catC* (muconolactone isomerase), and *pcaD* (3-oxoadipate enol-lactonase) (Table S1d), ultimately converting catechol into acetyl-CoA and succinyl-CoA, which feed into the TCA cycle. In addition, we detected a gene encoding the quercetin-2,3-dioxygenase (QueD; Table S1d) for oxidative cleavage of quercetin, a plant-derived flavonoid (48). Pathways for metabolism of aromatic compounds, including toluene, o-cresol, m-methylbenzoic acid, benzoate, and benzene, were also present. For toluene, we detected the genes *dmpL*, *dmpM*, *dmpN*, *dmpO*, *dmpP*, and *dmpK*, which encode components of the phenol hydroxylase complex for hydroxylation of toluene to o-cresol. O-cresol is further oxidized to 2,3-dihydroxytoluene before ring cleavage (Table S1d), yielding intermediates such as acetyl-CoA and succinyl-CoA that enter the TCA cycle. For *m*-methylbenzoic acid and benzoate, we detected the genes *benA*, *benB*, and *benC*, which encode the benzoate/toluate 1,2-dioxygenase complex that catalyzes the initial dihydroxylation of the aromatic ring to form cis-dihydrodiol intermediates (Table S1d). These intermediates are subsequently oxidized by *benD* (dihydroxycyclohexadiene carboxylate dehydrogenase) to yield 3-methylcatechol from *m*-methylbenzoate and catechol from benzoate. Both catechol and methylcatechol then enter ring-cleavage pathways (catechol via the β-ketoadipate pathway involving *catA*, *catB*, *catC*, and *pcaD*, and methylcatechol through the meta-cleavage route), ultimately producing acetyl-CoA and succinyl-CoA for entry into the TCA cycle. Finally, for benzene, we detected the *dmp* phenol hydroxylase gene cluster (*dmpL*, *dmpM*, *dmpN*, *dmpO*, *dmpP*, and *dmpK*; Table S1d), which yields enzymes involved in the sequential hydroxylation of benzene to phenol and then to catechol. Catechol is subsequently processed as described above, ultimately yielding acetyl-CoA and succinyl-CoA that enter the tricarboxylic acid cycle. The phenols and aromatic compounds can be produced through a variety of industrial processes, including petroleum refining, herbicide manufacturing, coke production, olive oil processing, and petrochemical operations. The results suggest that *A. towneri* RMS-02 has potential for treating wastewater containing phenolics and aromatics.

The genome of *Acinetobacter towneri* RMS-02 harbors genes encoding secreted proteases and exopeptidases for the degradation of proteins and short peptide chains, suggesting some levels of protein breakdown in the waste sludge environment. Specifically, secreted serine aminopeptidases belonging to the MEROPS family S33 and metalloproteases from families M28 and M23 were detected (Table S1e). Identified transporters included a putative amino acid transporter, an amino acid permease, a serine/threonine transporter, a branched-chain amino acid:cation transporter of the LIVCS family, and a glycine transporter (Table S1f). For lipid metabolism, genes encoding predicted lipases, phospholipases, triacylglycerol lipases, and carboxylesterases, as well as transporters for long-chain fatty acids, were detected (Table S1g and h).

Next, we screened the *A. towneri* RMS-02 genome with an in-house–built database to identify genes encoding enzymes potentially involved in the degradation of biopolymers or chemical structures related to hydrocarbon-based synthetic polymers (e.g., alkanes). This led to the identification of several genes involved in alkane degradation, particularly those involved in the initial step of terminal hydroxylation (Table S1i). Relevant genes included *alkB*, coding for a membrane-associated alkane monooxygenase AlkB, previously reported to typically oxidize C10–C16 *n*-alkanes, although some versions can hydroxylate up to C32 *n-*alkanes (49, 50). A gene encoding AlkM, which can hydroxylate long-chain (C16–C22) and very long-chain alkanes (>C22), was also detected (51). The third type of alkane hydroxylase identified in the genome of *A. towneri* RMS-02 is the flavoprotein AlmA. This enzyme is thought to be involved in the terminal hydroxylation of alkanes with >C22 (52, 53), and it has also been linked to subterminal oxidation of alkanes (54). The presence of the different alkane hydroxylases in the genome of *A. towneri* RMS-02 indicates that the strain has a broad alkane degradation capacity. There was only one BlastP hit for a plastic-degrading enzyme: an arylesterase (Table S1i). Such enzymes have been implicated in the degradation of the biodegradable copolyester PBAT (55).

Overall, the results show the biodegradative potential of *Acinetobacter towneri* RMS-02 toward phenolic and aromatic compounds, as well as short hydrocarbons, which are likely abundant in the waste sludge environment from which the strain was sourced.

### *A. towneri* RMS-02 produces enzymes enabling utilization of alkanes and ketones derived from an oxidized polyethylene substrate

**Fig 2:**
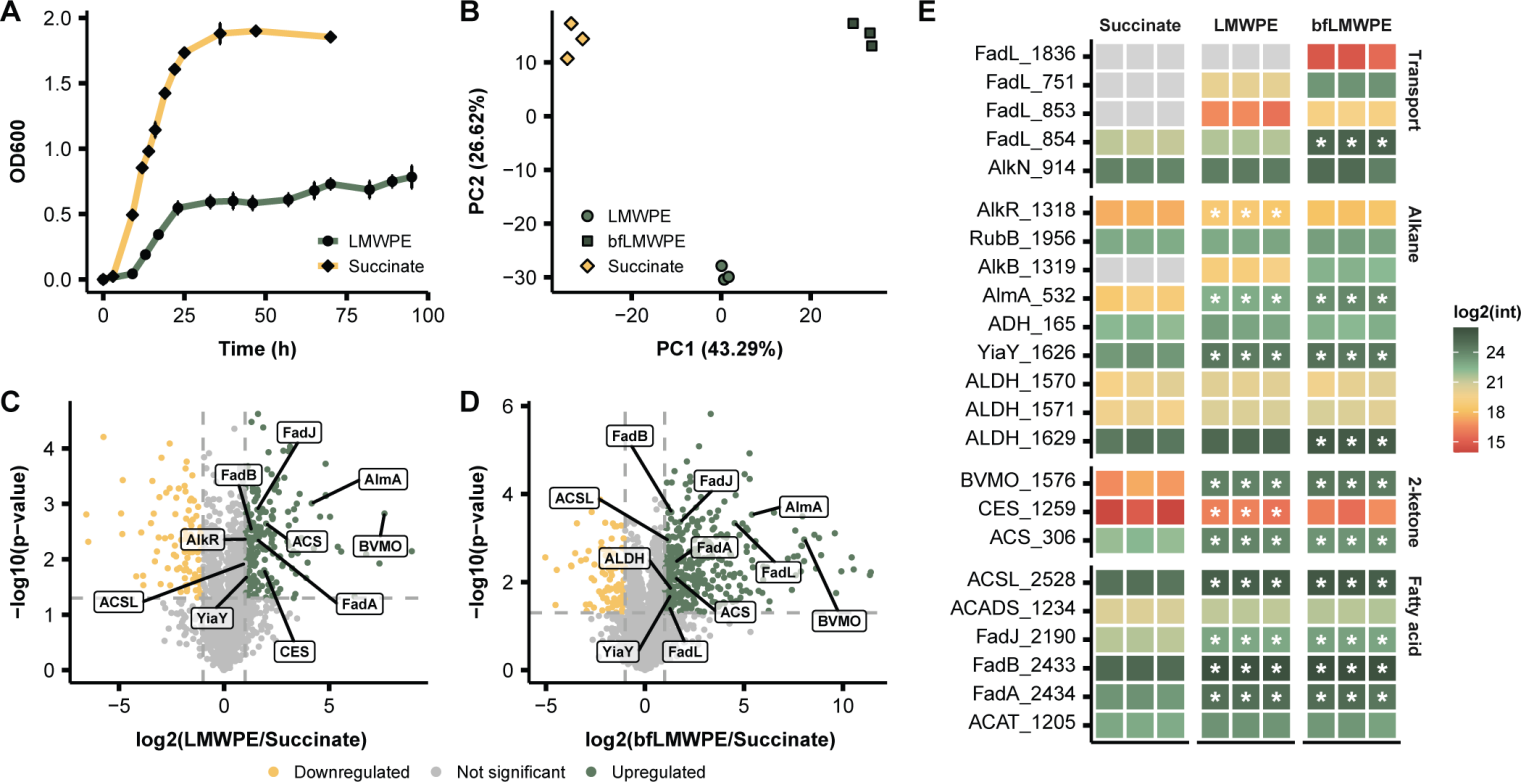
Substrate-dependent growth of *A. towneri* RMS-02 and differentially abundant proteins detected in the LMWPE proteomes derived from planktonic cells and biofilm. (**A**) Growth profiles for *A. towneri* RMS-02 on LMWPE or sodium succinate as the sole carbon source. (**B**) PCA analysis of *A. towneri* RMS-02’s proteomes obtained from both planktonic cells (LMWPE) and biofilm forming cells (bfLMWPE) during growth on LMWPE, as well as from cell growth on sodium succinate. PCA demonstrates clear separation between groups with tight clustering of biological replicates. (**C**) Volcano plot comparing proteomes of planktonic cells derived from cultures on LMWPE and sodium succinate. (**D**) Volcano plot comparing proteomes of biofilm-derived cells (bfLMWPE) to planktonic cells (LMWPE) from LMWPE cultures. In both panels C and D, the dashed horizontal line denotes the *P*-value cutoff (*P* < 0.05), while the dashed vertical lines denote the log_2_-fold change cutoff (< −1 and > 1). (**E**) Heatmap of selected proteins involved or hypothesized to be involved in the transport of fatty acids/2-ketones, alkane degradation, 2-ketone degradation, and fatty acid metabolism. Stars indicate proteins that are significantly differentially abundant (*P* < 0.05, −1 > log_2_-fold change > 1) when comparing LMWPE or bfLMWPE to sodium succinate. Source data can be found in Table S2.

To identify enzymes responsible for the degradation of short hydrocarbons, we performed a proteomic analysis of planktonic and biofilm-associated proteins produced by *A. towneri* during growth on sodium succinate or on a substrate that resembles an abiotically oxidized form of PE (referred to as LMWPE, Sigma-Aldrich, product number 427772). This substrate has previously been shown to include a polymeric LMWPE fraction, as well as medium- to long-chain alkanes and their oxidized variants (18). To compare the proteomes under two different growth conditions, *A. towneri* RMS-02 cells were collected at mid-exponential phase from cultures grown on LMWPE and sodium succinate (Fig. 2A). Principal component analysis (PCA) of the LMWPE, LMWPE biofilm (bfLMWPE), and sodium succinate proteome indicated that samples from each condition exhibited distinct clustering patterns, indicating consistent proteomic profiles within conditions and clear separation between clusters, reflecting substantial differences in overall protein expression patterns between substrates (Fig. 2B).

To gain insight into the enzymes and pathways used by *A. towneri* RMS-02 during growth on LMWPE, we compared its proteomic profile under this condition to that observed during growth on sodium succinate. Most enzymes involved in both alkane degradation and β-oxidation were found to be differentially abundant in the LMWPE proteome (Fig. 2C and D). These include AlkR, a transcriptional regulator of alkane hydroxylase (56), AlmA, and the alcohol dehydrogenase YiaY (KO ID: K13954). AlkB was not detected in the sodium succinate proteome, but it was exclusively found in the LMWPE and bfLMWPE proteome (Fig. 2E). Other detected enzymes known to be involved in the alkane degradation pathway that were more abundant in the LMWPE-derived proteome include a rubredoxin-NAD(+) reductase (RubB), an alcohol dehydrogenase (ADH), and three aldehyde dehydrogenases (ALDH) (Fig. 2E).

A putative Baeyer–Villiger monooxygenase (BVMO; BVMO_1576), a carboxylesterase (CES_1259), and an acetyl-CoA synthetase (ACS_306) were significantly more abundant in the LMWPE-derived proteomes (Fig. 2C through E). BVMOs are a class of flavin monooxygenases that catalyze the insertion of an oxygen between carbons in carboxyl groups (57). BVMOs are involved in the subterminal oxidation of alkanes (58), where they oxidize carbonyl groups into an ester (14). Because LMWPE contains 2-ketones (18), a BVMO is necessary for conversion into an ester. An esterase, such as CES_1259, can then hydrolyze the ester into a primary alcohol and an acetate, which can enter into the terminal alkane degradation pathway and the tricarboxylic acid (TCA) cycle, respectively (59). The detection of the BMVO_1576, CES_1259, and ACS_306 at higher levels in the LMWPE proteomes suggests their involvement in the oxidation of 2-ketones present in the substrate. After terminal and subterminal oxidation of alkanes or 2-ketones, the resulting compounds enter the β-oxidation pathway. Most of the enzymes involved in the β-oxidation pathway were also significantly differentially abundant in both the LMWPE and the bfLMWPE proteomes (Fig. 2E), including long-chain fatty-acyl-CoA ligase (ACSL_2528), acyl-CoA dehydrogenase (ACADS_1234), enoyl-CoA hydratase/3-hydroxyacyl-CoA dehydrogenase (FadJ_2190), 3-hydroxyacyl-CoA dehydrogenase (FadB_2433), β-ketothiolase (FadA_2434), and acetyl-CoA C-acetyltransferase (ACAT_1205).

Depending on the alkane hydroxylase, alkanes are either transported into the cell during hydroxylation (via AlkB) or need to be transported independently into the cell prior to degradation (via AlmA). In the LMWPE proteome, there are several transporters of hydrophobic compounds (FadL) which are not detected in the sodium succinate proteome (Fig. 2E). FadL is known to facilitate the transport of fatty acid across the outer membrane of Gram-negative bacteria, and these transport proteins might be involved in the uptake of other hydrophobic substances and xenobiotics (60). As LMWPE contains both 2-ketones and alkanes, this suggests that FadL transporter could be involved in the uptake of these molecules into the bacterial cell prior to depolymerization.

Both LMWPE and bfLMWPE proteomes showed differentially abundant proteins associated with transport of fatty acids and alkanes, the degradation of alkanes and 2-ketones, and β-oxidation (Fig. 2C through E). There are, however, some differences in these proteomes, such as significantly differentially abundant proteins in the bfLMWPE proteome associated with biofilm formation. These include phospholipid-binding lipoprotein (MlaA; AtRMS-02_contig_1_738), biofilm PGA synthesis protein (PgaA; AtRMS-02_contig_1_2022), and polysaccharide biosynthesis/export protein (AtRMS-02_contig_1_89) (Table S2).

Overall, analysis of the differentially abundant proteins in the LMWPE-derived proteomes indicates that *A. towneri* RMS-02 harnesses its complement of alkane- and 2-ketone–degrading enzymes, channeling these substrates through the β-oxidation pathway. This underscores the isolate’s metabolic versatility and its potential to thrive on hydrocarbon contaminants commonly found in waste sludge environments.

### *A. towneri* RMS-02 metabolizes alkanes and 2-ketones but does not degrade high molecular weight PE

**Fig 3:**
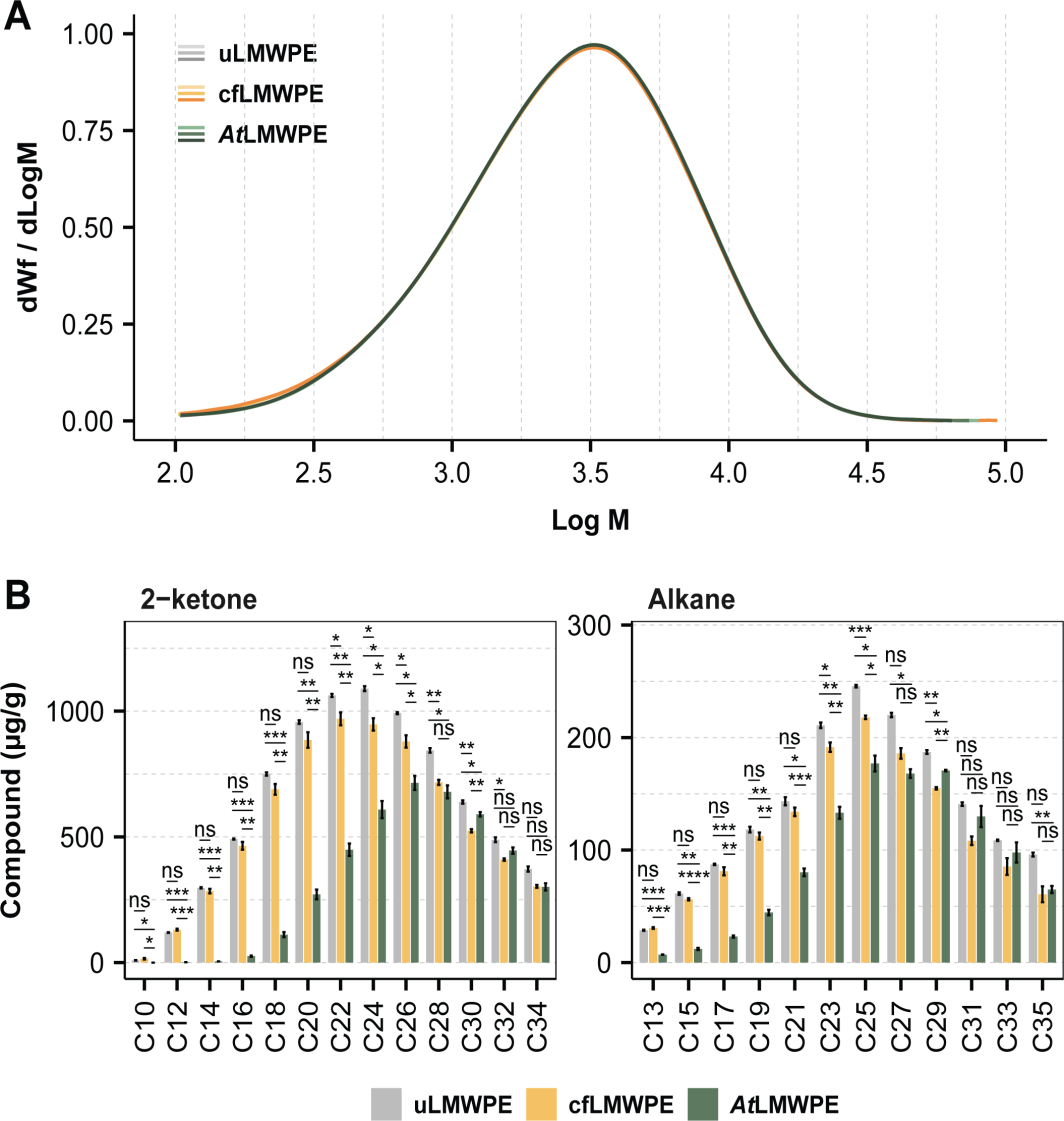
Characterization of LMWPE post-degradation with *A. towneri* RSM-02 and compositional profiles of abiotic controls. The isolate was grown with 30 mg/mL LMWPE in MM (*At*LMWPE). Controls included LMWPE in cell-free MM (cfLMWPE) and untreated LMWPE (uLMWPE). (**A**) SEC chromatograms of the samples uLMWPE, cfLMWPE, and *At*LMWPE. The chromatograms are superimposing, both for samples and replicates, in the high-molar-mass region, indicating no polymer degradation has occurred. Lines in the legend for each condition represent biological triplicates. (**B**) GC–MS results show levels of 2-ketones (left panel) and alkanes (right panel) in LMWPE extracts. Data are means ± standard deviation of three biological replicates. Significance was determined using a pairwise *t*-tests: not significant (ns), *P* ≤ 0.05 (*), *P* ≤ 0.01 (**), *P* ≤ 0.001 (***), and *P* ≤ 0.0001 (****). Source data for SEC and GC–MS are available in Table S3.

We conducted a detailed analysis of the LMWPE substrate before and after bacterial growth to identify the constituents supporting the growth of *A. towneri* RMS-02. Members of the genus *Acinetobacter* have previously been reported to degrade hydrocarbons, from alkanes to PE (9, 61, 62), and enzymes involved in the alkane degradation pathway have also been linked to the degradation of PE (63). Of note, while previous literature has concluded that *Acinetobacter* spp. can depolymerize PE, these investigations used the same LMWPE as the substrate for growth (64, 65). Nevertheless, the substrate was not characterized in a way that would clarify whether the high *M*_*w*_ component itself was utilized, or if the observed growth was limited to the utilization of PE-derived constituents, including alkanes and ketones, from possible abiotic oxidation. To assess whether *A. towneri* RMS-02 could depolymerize the high *M*_*w*_ PE fraction of LMWPE, SEC was conducted on the substrate after bacterial growth. When comparing chromatograms of LMWPE incubated with the isolate (*At*LMWPE) to LMWPE incubated in cell-free MM (cfLMWPE) and untreated LMWPE (uLMWPE), the profiles for all samples were virtually superimposing, indicating no alteration of the polymeric fraction (Fig. 3A). There was no reduction in molar mass distribution in the *At*LMWPE samples, which would have occurred if the strain was able to degrade PE. Although the PE component remained unchanged, *A. towneri* RMS-02 effectively depleted the alkanes and 2-ketones found in LMWPE (Fig. 3B). Specifically, the isolate was able to degrade *n-*alkanes ranging from C13 to C25 as well as 2-ketones of C10–C26. This is in accordance with the differentially abundant proteins found in the LMWPE proteome, including AlkB, AlmA, and a putative BVMO (Fig. 2C through E).

### Conclusion

**Fig 4:**
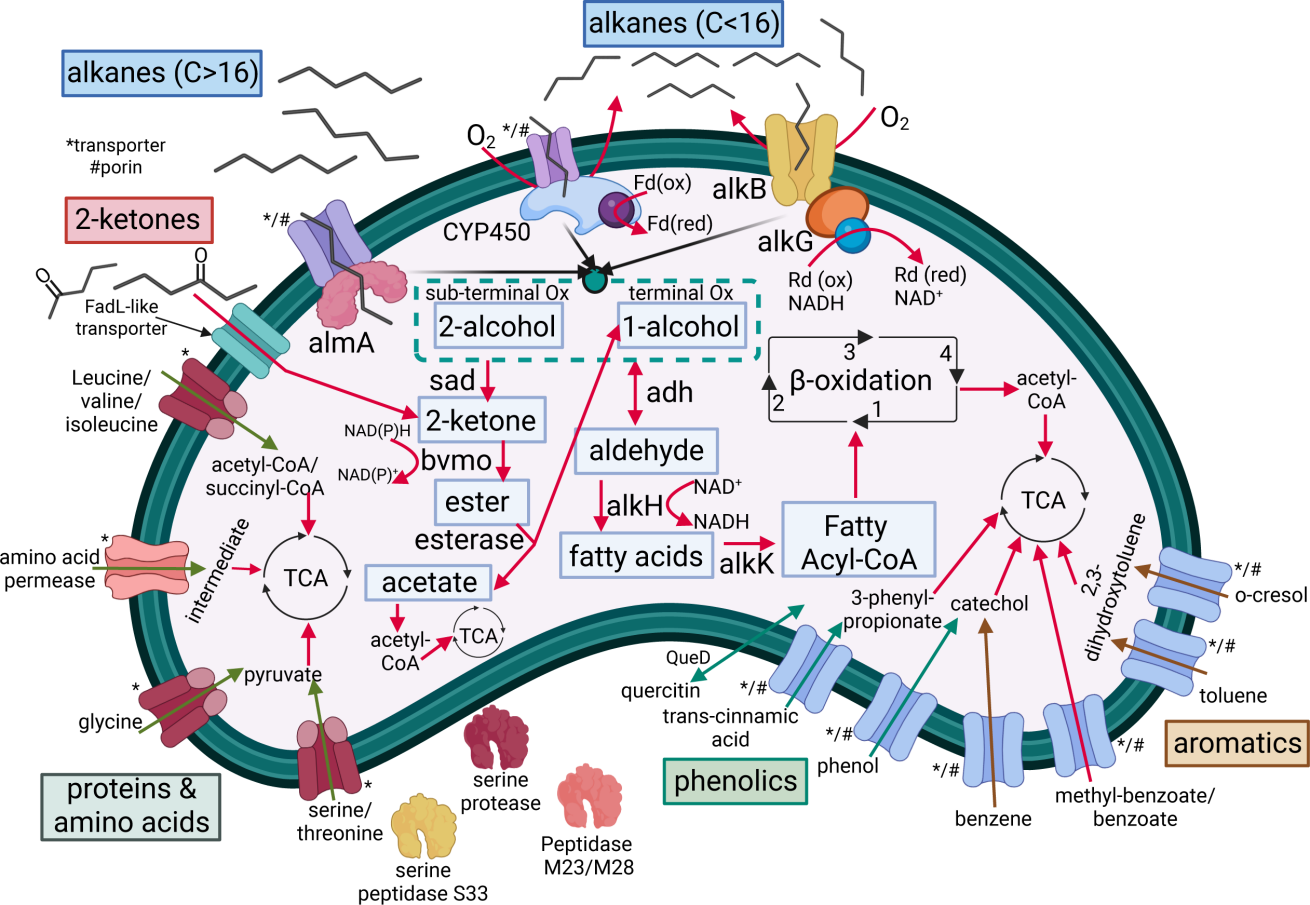
Schematic overview of selected metabolic pathways detected in *A. towneri* RMS-02 based on genomic predictions and proteomics evidence. Abbreviations: almA, long-chain alkane monooxygenase; CYP450, cytochrome P450 monooxygenase; Fd(ox), oxidized ferredoxin; Fd(red), reduced ferredoxin; alkB, alkane hydroxylase B; Rd(ox), oxidized rubredoxin; Rd(red), reduced rubredoxin (electrons provided from NADH); sub-terminal Ox, pathway for sub-terminal oxidation of alkanes; terminal Ox, pathway for terminal oxidation of alkanes; sad, secondary alcohol dehydrogenase; bmvo, Baeyer–Villiger monooxygenase; adh, alcohol dehydrogenase; alkH, aldehyde dehydrogenase; alkK, acyl-CoA synthetase; enzymes in fatty acid β-oxidation of fatty acids are 1, acyl-CoA oxidase or acyl-CoA dehydrogenase; 2, enoyl-CoA dehydratase; 3, 3-hydroxyacyl-CoA dehydrogenase; 4, β-ketoacyl-CoA thiolase; TCA, tricarboxylic acid cycle, QueD, quercetin-2,3-dioxygenase; FadL, long-chain fatty acid transporter. A * or a # indicates a transporter or a porin, respectively.

In this study, we reveal the previously unrecognized metabolic potential of *A. towneri* RMS-02 for the degradation of diverse compounds and contaminants found in wastewater sludge. Genomic analysis identified a broad repertoire of genes encoding enzymes for the utilization of polyphenols and aromatic compounds. Genes coding for enzymes enabling degradation of proteins and peptides, as well as transporters for fatty acids, were also detected, suggesting that this isolate can access multiple nutrient sources commonly found in wastewater. Integrating genomic and proteomic analyses with comprehensive characterization of the LMWPE substrate before and after bacterial growth, we demonstrate that the isolate is capable of degrading both medium- and long-chain alkanes (C13–C25), as well as 2-ketones of C10–C26. This finding reveals a metabolic capability that, to our knowledge, has not been previously documented for *A. towneri*, a species so far known only to degrade phenols (17). Although *A. towneri* RMS-02 successfully degraded LMWPE derivatives, it does not possess enzymes to break down the high *M*_*w*_ component of the substrate. However, in wastewater sludge (66), microplastics can persist and undergo abiotic oxidation through exposure to oxygen, elevated temperatures, and chemical oxidants, generating smaller oxidized fragments such as ketones and alcohols (67). The capacity of *A. towneri* RMS-02 to utilize the oxidized LMWPE derivatives, as well as its genomic potential for polyphenol and aromatic degradation (Fig. 4), highlights the possible use of this isolate or its enzymes in bioremediation of wastewater sludge. Further research should focus on the biochemical characterization of the enzymes involved in the degradation of PE derivatives, which could be used to develop enzymatic cocktails tailored to the removal of alkanes and ketones derived from crude oil or oxidized plastics in contaminated environments.

## Data Availability

Long-read DNA sequences have been deposited in the European Nucleotide Archive under the project accession number PRJEB103840. The genome sequence, gene annotations, and predicted proteins have been made publicly available via FigShare (https://doi.org/10.6084/m9.figshare.30427936). The mass spectrometry proteomics data has been deposited to the ProteomeXchange Consortium via the PRIDE (Proteomics Identification Database) partner repository with the data set identifier PXD069492. Scripts used to generate the phylogenomic tree, can be found at githubGitHub (https://github.com/ronjasan/AtRMS-02). All data are publicly available and comply with the data reuse guidelines presented in Hug et al. (68).

## SUPPLEMENTAL MATERIAL

Table S1 spectrum.00054-26-s0001.xlsx Genes encoding CAZymes, enzymes for metabolism of polyphenols and aromatics, proteolytic enzymes, amino acid transporters, lipases, fatty acid transporters, and alkane degrading enzymes detected in the genome of *A. towneri* RMS-02.

Table S2 spectrum.00054-26-s0002.xlsx Source data for proteomics.

Table S3 spectrum.00054-26-s0003.xlsx SEC and GC-MS data for LMWPE post-incubation with *A. towneri* RMS-02.

ASM does not own the copyrights to Supplemental Material that may be linked to, or accessed through, an article. The authors have granted ASM a non-exclusive, world-wide license to publish the Supplemental Material files. Please contact the corresponding author directly for reuse.
